# Is contraceptive self-injection cost-effective compared to contraceptive injections from facility-based health workers? Evidence from Uganda^☆^^☆☆^

**DOI:** 10.1016/j.contraception.2018.07.137

**Published:** 2018-11

**Authors:** Laura Di Giorgio, Mercy Mvundura, Justine Tumusiime, Chloe Morozoff, Jane Cover, Jennifer Kidwell Drake

**Affiliations:** a PATH, PO Box 900922, Seattle, WA 98109, USA; b PATH, PO Box 7404, Kampala, Uganda

**Keywords:** Cost-effectiveness, Economic evaluation, DMPA-SC, Injectable contraception, Self-injection, Family planning

## Abstract

**Objective:**

To assess the cost-effectiveness of self-injected subcutaneous depot medroxyprogesterone acetate (DMPA-SC) compared to health-worker-administered intramuscular DMPA (DMPA-IM) in Uganda.

**Study design:**

We developed a decision-tree model with a 12-month time horizon for a hypothetical cohort of approximately 1 million injectable contraceptive users in Uganda to estimate the incremental costs per pregnancy averted and per disability-adjusted life year (DALY) averted. The study design derived model inputs from DMPA-SC self-injection continuation and costing research studies and peer-reviewed literature. We calculated incremental cost-effectiveness ratios from societal and health system perspectives and conducted one-way and probabilistic sensitivity analyses to test the robustness of results.

**Results:**

Self-injected DMPA-SC could prevent 10,827 additional unintended pregnancies and 1620 maternal DALYs per year for this hypothetical cohort compared to DMPA-IM administered by facility-based health workers. Due to savings in women's time and travel costs, under a societal perspective, self-injection could save approximately US$1 million or $84,000 per year, depending on the self-injection training aid used. From a health system perspective, self-injection would avert more pregnancies but incur additional costs. A training approach using a one-page client instruction sheet would make self-injection cost-effective compared to DMPA-IM, with incremental costs per pregnancy averted of $15 and per maternal DALY averted of $98. Sensitivity analysis showed that the estimates were robust. The one-way and probabilistic sensitivity analyses showed that the costs of the first visit for self-injection (which include training costs) were an important variable impacting the cost-effectiveness estimates.

**Conclusions:**

Under a societal perspective, self-injected DMPA-SC averted more pregnancies and cost less compared to health-worker-administered DMPA-IM. Under a health system perspective, self-injected DMPA-SC can be cost-effective relative to DMPA-IM when a lower-cost visual aid for client training is used.

**Implications:**

Self-injection has economic benefits for women through savings in time and travel costs, and it averts additional pregnancies and maternal disability-adjusted life years compared to health-worker-administered injectable DMPA-IM. Implementing lower-cost approaches to client training can help ensure that self-injection is also cost-effective from a health system perspective.

## 1 Introduction

Investments in satisfying unmet need for contraception—thereby preventing unintended pregnancies, unplanned births and induced abortions—reduce maternal morbidity and mortality. Investing in contraceptive services in addition to maternal and newborn services in low- and middle-income countries could save nearly US$7 billion compared with investing in maternal and newborn services alone [1].

Previous analyses have shown that any modern contraceptive is cost-saving compared to no contraception [2], [3], [4]. However, the literature on the relative cost-effectiveness of different modern contraceptive methods in low-resource settings is less conclusive. Most analyses have been conducted in high-income countries [2], [3], [4], [5], [6], [7], [8], [9], while evidence from developing countries remains scarce [10], [11]. Not surprisingly, perhaps, most analyses indicate that sterilization and long-acting reversible contraceptive methods (e.g., copper T intrauterine device, intrauterine system, contraceptive implant) are the most cost-effective family planning alternatives [12]; however, these are not always women's preferred methods [10], [11], and they depend on availability of skilled health workers, which can be limited in low-resource settings.

Among women using contraception in Uganda, where overall unmet need remains high, the most common method is the injectable [13]. Subcutaneous depot medroxyprogesterone acetate (DMPA-SC) is a novel injectable contraceptive that can be self-administered by women after training with a health worker [14]. Self-injection eliminates the need for quarterly visits to the clinic, which has the potential to reduce a common reason for discontinuation of injectables: being late for injection [15]. Previous studies demonstrate that self-injection with DMPA-SC is feasible and highly acceptable [16], [17]. In addition, newly published research in Uganda, Malawi, and the United States demonstrates that women who self-injected DMPA-SC had higher 12-month continuation rates than women who received DMPA from health workers [18], [19], [20]. However, the cost-effectiveness of self-injection compared to health-worker-administered injections has not been evaluated. This study aims to fill this research gap by exploring cost-effectiveness of self-injected DMPA-SC compared to health-worker-administered DMPA-IM in Uganda.

The Uganda continuation study referenced above [18] provided a unique opportunity to assess the cost-effectiveness of self-injection. The study used a prospective cohort design, where women self-injecting DMPA-SC and women receiving DMPA-IM from a facility-based health worker were interviewed and followed every 3 months to estimate continuation rates at 12 months (81% among self-injectors and 65% among DMPA-IM users). We conducted the continuation study alongside a costing study that collected primary costing data to estimate the health system costs of delivering the injectables [21]. Study staff obtained data on women's time and travel costs from interviews with the women included in the continuation studies.

Information on the economic costs and corresponding benefits of various contraceptive options and delivery strategies can help decision-makers, implementers, civil society groups and advocates make evidence-based decisions about family planning policy and programs. The objective of this study was to assess the cost-effectiveness of self-injected DMPA-SC compared to health-worker-administered DMPA-IM and provide evidence on whether the benefits of self-injection (as demonstrated by longer continuation rates and hence fewer unintended pregnancies) are worth any additional costs compared to health-worker-administered DMPA-IM in Uganda.

## 2 Methods

### 2.1 Comparison of DMPA delivery strategies

We compared self-injection of DMPA-SC (delivered within the context of the research study conducted in Uganda [18]) to facility-based health worker administration of DMPA-IM. Under the research study, women opting to receive an injectable contraceptive at a health facility chose to either self-inject DMPA-SC or receive DMPA-IM from a facility-based health worker. Women who chose DMPA-IM had the injection administered by the health worker, and study staff asked them to return to the facility every 3 months for their next injection. Study nurses used water-filled devices to train clients who chose self-injection and gave each woman a calendar to assist with reinjection dates and an instruction booklet as a client training aid. Clients self-injected for the first time at the health facility under the supervision of the health worker. Those deemed proficient took three doses home for independent self-injection and were advised to dispose of used injection devices in a latrine. Researchers followed up with clients to measure continuation rates (the measure of “effectiveness” employed in the cost-effectiveness analysis) at 12 months (after four injections) for the two delivery strategies.

To adapt the research intervention to better reflect the current standard of practice for self-injection in Uganda, we substituted the training booklet for a one-page instruction sheet and considered that women were given a disposal container for storing used injection units until they could be returned to a health facility or health worker. The one-page (two-sided) instruction sheet currently used in programmatic implementation contains the same information as the booklet used in the research study. We assumed that staff provided the impermeable, low-cost disposal containers for storing used injection units free of charge to women. The cost analysis reflects the implications of both adaptations (i.e., reduced cost for the client training aid and small additive cost for the impermeable containers); we did not expect either adaptation to change women's ability to self-inject correctly or impact continuation.

### 2.2 Overview of the cost-effectiveness model

**Fig. 1:**
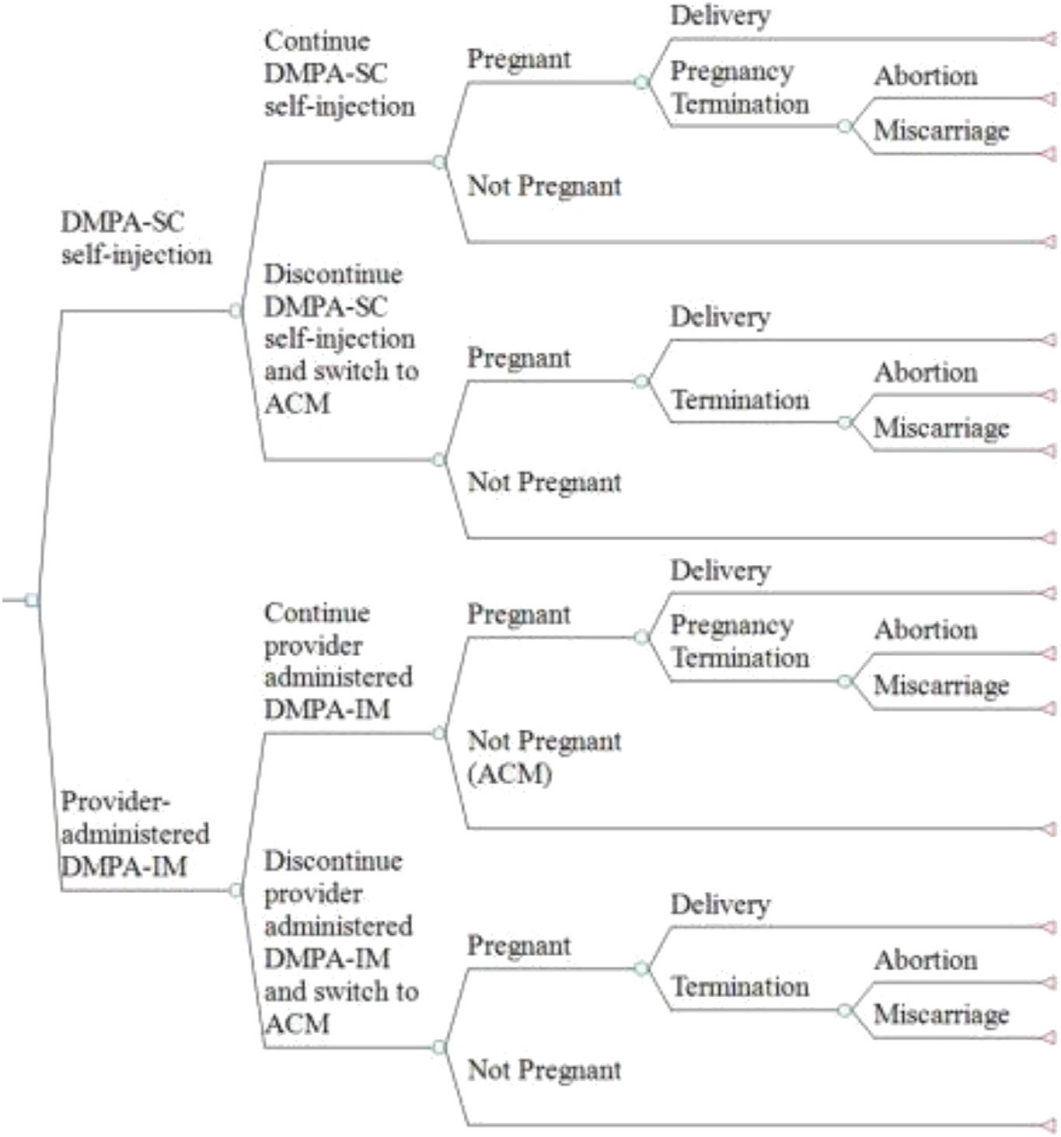
Decision-tree model to compare the costs and effectiveness of self-injected DMPA-SC versus health-worker-administered DMPA-IM.

We used a decision-tree model to evaluate the outcomes of continuation and discontinuation of either DMPA-SC or DMPA-IM (Fig. 1) for a hypothetical cohort of approximately 1 million Ugandan women using injectable contraceptives. The cohort size reflects the estimated number of women of reproductive age in Uganda who used injectable contraceptives in 2015 [22], [23]. We then allocated this number equally between self-injection of DMPA-SC and receipt of DMPA-IM from a health worker. As described in Fig. 1, after self-injecting DMPA-SC or receiving DMPA-IM from a health worker, each woman could choose to either continue using the injectable or discontinue. Women who continue or discontinue would then either become pregnant or not. Each pregnancy would result in a delivery or pregnancy termination (miscarriage or abortion). We modeled a 1-year time horizon to reflect the injectable continuation duration used in the study and assumed that any woman who discontinued the method did so at 6 months. In the event of discontinuation, we assumed that women discontinued using contraception altogether or switched to another contraceptive method (modern or traditional) or no method. We used the average contraceptive method (ACM) approach to model the effectiveness and the costs of the method to which they switched [7]. The ACM approach weighted the average contraceptive costs and effectiveness according to each injectable group's switching behavior. The proportions of women switching to each contraceptive method or no method differed by injectable group, and we based these on data from the self-injection research study [18]. Women who discontinued self-injection could also choose to receive DMPA-SC injections from a health worker. We based the costs of these DMPA-SC injections on the costs of DMPA-IM administered at health facilities, adjusted for the slightly higher commodity price of DMPA-SC.

### 2.3 Model data inputs

We ran the analysis from both the health system and societal perspectives. The health system perspective accounted for the direct medical costs of providing injectable contraceptives: for contraceptive commodities, health worker time for service delivery, supplies and tests, drugs used to treat side effects and health facility waste disposal. We derived data on the costs of contraceptive service delivery from a microcosting study (Table 1) [21]. We included additional costs for self-injection training supplies and health worker time to provide training for DMPA-SC users. Under a societal perspective, we added women's travel and time costs to receive contraception (DMPA-SC, DMPA-IM or the contraceptives to which they switched in the case of injectable discontinuation) (Table 1). Since the analysis had a 1-year time horizon, we assumed that women who continued for 1 year would use four units of DMPA.

**Table 1:** Key cost inputs, per client (in 2016 US$)

Parameter	Base case	Data source	Minimum; maximum; For one-way sensitivity analysis^d^
Costs under the health system perspective			
Direct medical costs of DMPA-SC self-injection for 4 injections	$8.11/6.35^c^	Di Giorgio et al., 2018 [21]	−
Direct medical costs for first visit for DMPA-SC self-injection at the health facility^a^	$5.44/3.68^c^	Di Giorgio et al., 2018 [21]	$2.50^c^; $10.88
Direct medical costs for each subsequent DMPA-SC self-injection away from the facility	$0.89	Di Giorgio et al., 2018 [21]	$0.85; $1.78
Direct medical costs of health-worker-administered DMPA-IM for 4 injections	$5.46	Di Giorgio et al., 2018 [21]	−
Direct medical costs for first DMPA-IM injection by a facility-based health worker^a^	$1.65	Di Giorgio et al., 2018 [21]	$0.83; $3.30
Direct medical costs for each subsequent DMPA-IM injection by a facility-based health worker	$1.27	Di Giorgio et al., 2018 [21]	$0.83; $2.16
Direct medical costs of the ACM for 0.5 year after discontinuing DMPA-SC	$1.20	Di Giorgio et al., 2018 [21]	$0.60; $2.40
Direct medical costs of the ACM for 0.5 year after discontinuing DMPA-IM	$0.64	Di Giorgio et al., 2018 [21]	$0.32; $1.28
Costs under the societal perspective			
Direct medical and direct nonmedical costs of DMPA-SC self-injection for 4 injections	$9.72/$7.96^c^	Di Giorgio et al., 2018 [21]	−
Direct medical and direct nonmedical costs for first visit for DMPA-SC self-injection at the health facility	$6.78/$5.02^c^	Di Giorgio et al., 2018 [21]	$3.39; $10.88
Direct medical and direct nonmedical costs for each subsequent DMPA-SC self-injection away from the facility	$0.98	Di Giorgio et al., 2018 [21]	$0.85; $1.78
Direct medical and direct nonmedical costs of health-worker-administered DMPA-IM for 4 injections	$10.12	Di Giorgio et al., 2018 [21]	−
Direct medical and direct nonmedical costs for first DMPA-IM injection by a facility-based health worker	$2.77	Di Giorgio et al., 2018 [21]	$0.83; $6.38
Direct medical and direct nonmedical costs for each subsequent DMPA-IM injection by a facility-based health worker	$2.45	Di Giorgio et al., 2018 [21]	$0.83; $3.85
Direct medical and direct nonmedical costs of the ACM for 0.5 year after discontinuing DMPA-SC	$1.82	Di Giorgio et al., 2018 [21]	$0.91; $3.62
Direct medical and direct nonmedical costs of the ACM for 0.5 year after discontinuing DMPA-IM	$0.88	Di Giorgio et al., 2018 [21]	$0.44; $1.75
Direct medical costs of pregnancy			
Birth and newborn care costs^b^	$59.43	Babigumira et al., 2011 [34]	$29.72; $118.86
Miscarriage (between 12 and 22 weeks)	$2.58	Babigumira et al., 2011 [34]	$1.29; $5.16
Abortion	$88.94	Babigumira et al., 2011 [34]	$44.47; $177.88

a Includes medical examination costs; in the case of self-injection, includes training costs.

b Includes delivery, antenatal care, postnatal care and newborn care costs.

c The range is wide enough to include scenarios where a booklet is used as the training aid and also when the one-page instruction sheet is used.

d A lognormal distribution was used in the probabilistic sensitivity analysis.

We obtained 12-month continuation rates from a study conducted in Uganda: 81% for self-injectors and 65% for women receiving DMPA-IM from a health worker [18]. This study also provided information on the methods women chose after discontinuing DMPA, which we used as weights in the calculation of the ACM. We retrieved typical-use effectiveness data from the literature (Table 2) [24].

**Table 2:** Key inputs to estimate effectiveness, including contraceptive continuation rates and typical-use effectiveness

Indicator	Base case (rate)	Data source	Minimum and maximum values used in the sensitivity analysis^a^
Continuation rates			
12-month continuation rate with DMPA-SC self-injection	0.81	Cover et al., 2018 [18]	0.60; 0.95
12-month continuation rate with DMPA-IM	0.65	Cover et al., 2018 [18]	0.40; 0.85
Types of contraceptives to which women switched after discontinuing self-injection of DMPA-SC (among those who had already switched to another contraceptive or planned to do so within 30 days)			
Oral contraceptives	9	Cover, personal communication, 2017	See footnote^b^
Intrauterine device	9	Cover, personal communication, 2017	
DMPA-IM or DMPA-SC administered by a health worker	69	Cover, personal communication, 2017	
Implant	5	Cover, personal communication, 2017	
Male condoms	9	Cover, personal communication, 2017	
Traditional methods	0	Cover, personal communication, 2017	
Types of contraceptives to which women switched after discontinuing health-worker-administered DMPA-IM (among those who had already switched to another contraceptive or decided to switch)			
Oral contraceptives	5	Cover, personal communication, 2017	See footnote^c^
Intrauterine device	5	Cover, personal communication, 2017	
Other injectable administered by a health worker	5	Cover, personal communication, 2017	
Implant	20	Cover, personal communication, 2017	
Male condoms	55	Cover, personal communication, 2017	
Traditional methods	10	Cover, personal communication, 2017	
Cumulative effective rates [1−failure rate] of injectables and other contraceptives to which women switched after discontinuation (for 12 months of use in Uganda)			
Injectable effectiveness	95.6	Polis, 2016 [24]	90;97
Oral contraceptives	87.4	Polis, 2016 [24]	83;92
Intrauterine device	98.8	Polis, 2016 [24]	95;100
Implant	99.2	Polis, 2016 [24]	95;100
Male condoms	94.6	Polis, 2016 [24]	90;98
Traditional method (average of withdrawal and periodic abstinence)	82.1	Polis, 2016 [24]	73;87
Weighted average effectiveness of the ACM to which women switched			
ACM effectiveness (typical use) among women who discontinued self-injection of DMPA-SC	91.3	Calculated	85; 100
ACM effectiveness (typical use) among women who discontinued health-worker-administered DMPA-IM	87.3	Calculated	81; 92
Probability of pregnancy outcomes			
Probability of a delivery	71	Prada et al. 2016 [35]	See footnote^d^
Probability of a miscarriage	16	Prada et al. 2016 [35]	
Probability of an abortion	14	Prada et al. 2016 [35]	
Inputs for the DALY calculations			
YLL per maternal death (all causes)	56.499	Murray et al., 2010 [26]	NA
DALY ratio (YLD/YLL)	0.103	Murray et al., 2010 [26]	NA

Abbreviation: NA, not applicable.

a Beta distributions were assumed for the sensitivity analysis, with parameter values of *α*=2 and *β*=2.

b These percentages are correlated and add to 1. The sensitivity analysis focused on changing the most common method women switched to after discontinuing self-injection of DMPA-SC and adjusted the percentages for the other methods so that the total would still be 100%. In the low scenario, we assumed that less women would switch to injectables provided by a health worker and would switch to less effective methods. We assumed that 40% would use injectables and increased the percentages in the less effective methods. In the high-value scenario, we assumed that 70% of the women would switch to injectables provided by a health worker.

c Similar to the above, we modified the most common method used by women discontinuing health-worker-administered DMPA-IM. In the low scenario, we assumed that 20% of the women would switch to using condoms and more would opt for more effective methods. In the high-value scenario, we assumed that 70% of the women would switch to using condoms. Similarly, other percentages were adjusted such that the percentages add to 100%.

d These also add to 100% and so were varied at the same time. In the low scenario, we assumed 50% probability of a delivery, 34% abortions and 16% for miscarriage; in the high scenario, we assumed 75% for delivery, 5% abortions and 15% miscarriage.

In the case of DMPA discontinuation, we calculated 6 months of costs for the contraceptives to which women switched, including contraceptive commodity costs, health worker time for providing services and drugs for treating typical side effects. The analysis also included the costs of pregnancy-related outcomes under a health system perspective, such as prenatal care, delivery and pregnancy termination (Table 1); we obtained these costs from the literature and adjusted them using the inflation rate of 5% [25]. We included costs of pregnancy-related outcomes under the health system perspective because maternity care is free in Uganda. We did not account for the productivity costs associated with pregnancy or its outcomes.

### 2.4 Analysis

For each hypothetical cohort (women self-injecting DMPA-SC or receiving health-worker-administered DMPA-IM), we estimated the number of pregnancies and maternal disability-adjusted life years (DALYs) averted, the corresponding health system and societal costs of receiving the contraceptive services, and the costs associated with unintended pregnancies and their outcomes (delivery, miscarriage or abortion). We then estimated the incremental costs per pregnancy averted and per maternal DALY averted as the difference in costs divided by the difference in effectiveness (pregnancies or DALYs averted). We calculated the maternal DALYs averted for each pregnancy averted using the envelope approach based on data from the 2010 Global Burden of Disease Study for western sub-Saharan Africa [26]. We used 56.5 as the number of years of life lost (YLL) per maternal death (all causes); the ratio between years lost due to disability (YLD) and YLL was 0.103 for all maternal conditions. We then compared the incremental cost per DALY averted against a cost-effectiveness threshold for Uganda of $293/DALY averted [27]. This recently published and conservative threshold is lower than traditional thresholds provided by the World Health Organization, which are based on the GDP per capita: $615 in 2016 [28]. We conducted the analysis using Excel 2016 (Microsoft, Redmond, WA, USA).

### 2.5 Sensitivity analysis

We conducted one-way, two-way and probabilistic sensitivity analyses on all key inputs to explore the robustness of the results given the uncertainty of inputs. One-way sensitivity analysis evaluates how the cost per DALY averted changes when we change one model input at a time; we conducted this analysis in Excel using the minimum and maximum values shown in Table 1, Table 2. We allowed costs to vary — 50% to 200% from the mean. We conducted the two-way sensitivity analyses to investigate how the results differ when we assume that pairs of the five most influential model inputs identified in the one-way sensitivity analysis change at the same time. In addition, we conducted probabilistic sensitivity analysis using @Risk software (Palisades Corporation, Ithaca, NY, USA). Probabilistic sensitivity analyses are multiway sensitivity analysis using simulation methods. To do these, we assigned probability distributions to all key model inputs. We evaluated cost inputs assuming a lognormal distribution to account for skewness of costs [29] (Table 1) and assumed probabilities to follow a beta distribution (Table 2), as done in previous studies [30], [31]. We drew the set of key input values by randomly sampling from each distribution and ran the model 50,000 times to evaluate the robustness of the model estimates.

### 2.6 Ethical approval

This cost-effectiveness study used data from a costing study approved by the Mulago Research Ethics Committee of Uganda and a continuation study with ethical approval from PATH's Research Ethics Committee, the Mulago Research Ethics Committee of Uganda, and the Uganda National Council for Science and Technology. The approved study protocols specifically referenced the cost-effectiveness analysis.

## 3 Results

### 3.1 Base case analyses

For a hypothetical cohort of approximately 1 million women in Uganda, the higher continuation rates among women who self-inject DMPA-SC could result in averting an additional 10,827 pregnancies and 1620 maternal DALYs compared to health-worker-administered DMPA-IM (Table 3). When taking a societal perspective (i.e., including health system costs and women's travel and time costs), DMPA-SC would cost less than DMPA-IM. Self-injected DMPA-SC is therefore a dominant strategy from a societal perspective compared to health-worker-administered DMPA-IM (Table 3).

**Table 3:** Cost, effectiveness and incremental cost-effectiveness estimates for a hypothetical cohort of approximately 1 million injectable users in Uganda for a 1-year time horizon (in 2016 US$)

	Costs	Pregnancies averted	Maternal DALYs averted
Societal: research design			
DMPA-SC	$6,549,568	134,402	19,998
DMPA-IM	$6,633,425	123,575	18,378
Incremental	($83,857)	10,827	1620
Incremental cost-effectiveness ratio		Self-injected DMPA-SC is dominant	Self-injected DMPA-SC is dominant
Societal: programmatic implementation			
DMPA-SC	$5,632,352	134,402	19,998
DMPA-IM	$6,633,425	123,575	18,378
Incremental	($1,001,073)	10,827	1620
Incremental cost-effectiveness ratio		Self-injected DMPA-SC is dominant	Self-injected DMPA-SC is dominant
Health system: research design			
DMPA-SC	$5,667,770	134,402	19,998
DMPA-IM	$4,592,291	123,575	18,378
Incremental	$1,075,478	10,827	1620
Incremental cost-effectiveness ratio		$99/pregnancy averted	$664/DALY averted
Health system: programmatic implementation			
DMPA-SC	$4,750,553	134,402	19,998
DMPA-IM	$4,592,291	123,575	18,378
Incremental	$158,262	10,827	1620
Incremental cost-effectiveness ratio		$15/pregnancy averted	$98/DALY averted

Under the health system perspective, the total costs for self-injected DMPA-SC would be higher than the total costs for DMPA-IM, largely due to the costs of self-injection training during the first visit (Table 1). Total costs for 1 year for the cohort of women receiving health-worker-administered DMPA-IM were estimated at $4.6 million, while total costs for the cohort of women self-injecting DMPA-SC were estimated at approximately $4.8 million when using the one-page instruction sheet as a training aid and providing a disposal container. In this case, the incremental costs were estimated at $15 per pregnancy averted and $98 per maternal DALY averted (Table 3). Therefore, based on the upper end of the conservative incremental cost-effectiveness ratio thresholds for costs per DALY averted for Uganda ($293), under the programmatic implementation approach (with the one-page instruction sheet and disposal container) self-injection of DMPA-SC would be cost-effective compared to health-worker-administered DMPA-IM applying a health system perspective. When using the client instruction booklet and not providing a disposal container consistent with the research study approach, the total costs for 1 year would be $5.7 million (Table 3). The incremental cost per pregnancy averted was estimated at $99, while the incremental cost per maternal DALY averted was $664 in this case. This would not be considered as cost-effective when using a cost-effectiveness threshold of $293.

### 3.2 Sensitivity analysis

**Fig. 2:**
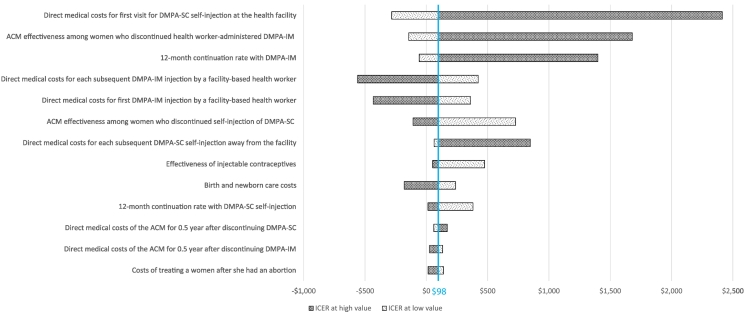
One-way sensitivity analysis for the health system perspective (under program design).

Most of our sensitivity results focus on the self-injection scenario where we consider the one-page instruction sheet as the client training aid, but we include wide ranges for the first visit costs which span the costs when the booklet is used as the training aid. The one-way sensitivity analysis (Fig. 2) showed that the model input that had the largest impact on the cost-effectiveness results was the direct medical costs of the first visit for self-injection training. Ranging this variable from $2.50 to $10.88 results in cost-effectiveness ranges from −$282 (dominant) to $2414 per DALY averted. Holding all other model inputs constant, when the costs for the first visit for self-injection training are below $3.40, self-injection is more effective and less costly (dominant) than provider-administered DMPA-IM. When the costs for the self-injection first visit are above $4.30, self-injection is not cost-effective (with $293 per DALY averted as the cost-effectiveness threshold). The second most influential model input was the effectiveness of the average contraceptive method that women switched to after discontinuing provider-administered DMPA-IM. Ranging this input from 81% to 92% contraceptive efficacy results in cost-effectiveness ranges from −$144 (dominant) to $1680. This means that if women who discontinue DMPA-IM tend to switch to methods that are more clinically effective, then self-injection is less likely to be cost-effective. The third most influential model input was the continuation rate for DMPA-IM administered by providers, and ranging this input from 0.4 to 0.81 results in cost-effectiveness ranges of −$38 (dominant) to $1399. Holding all other model inputs constant (including the continuation rate for self-injection at 0.81), if the continuation rate for DMPA-IM is above 0.75, then self-injection is not cost-effective using the threshold mentioned above.

The two-way sensitivity analysis results (Table 4) show the impact of changing pairs of the most influential model inputs identified in the one-way sensitivity analysis while holding all other inputs constant. The two-way sensitivity analyses show that if the costs for the first visit for self-injection training can be low, even if variables such as the continuation rate for provider administered DMPA-IM are high, self-injection can still be dominant or cost-effective. Also, if the methods that women who discontinue DMPA-IM switch to have high clinical effectiveness and continuation rates for DMPA-IM are high, then self-injection is likely to be less effective and less costly (dominated).

**Table 4:** Two-way sensitivity analyses results on the incremental cost per DALY averted for self-injection versus provider-administered DMPA-IM

Variable 1	Variable 2	Low of both variables 1 and 2	Low value of variable 1 and high of variable 2	High value of variable 1 low value of variable 2	High of both variables 1 and 2
Direct medical costs for first visit for DMPA-SC self-injection at the health facility	ACM effectiveness among women who discontinued health-worker-administered DMPA-IM	Dominant	$36	$993	$11,713
	12-month continuation rate with DMPA-IM	Dominant	Dominant	$1100	$2414
	Direct medical costs for first DMPA-IM injection by a facility-based health worker	Dominant	Dominant	$2678	$1883
	ACM effectiveness among women who discontinued self-injection of DMPA-SC	Dominant	Dominant	$6197	$1173
	Direct medical costs for each subsequent DMPA-SC self-injection away from the facility	Dominant	$468	$2380	$3164
	Direct medical costs for each subsequent DMPA-IM injection by a facility-based health worker	$44	Dominant	$2739	$1755
	Effectiveness of injectable contraceptives	Dominant	Dominant	$4771	$2207
ACM effectiveness among women who discontinued health-worker-administered DMPA-IM	12-month continuation rate with DMPA-IM	Dominant	$155	$584	Dominated
	Direct medical costs for first DMPA-IM injection by a facility-based health worker	Dominant	Dominant	$2823	Dominant
	ACM effectiveness among women who discontinued self-injection of DMPA-SC	Dominant	Dominant	Dominated	$91
	Direct medical costs for each subsequent DMPA-SC self-injection away from the facility	Dominant	$224	$1534	$4930
	Direct medical costs for each subsequent DMPA-IM injection by a facility-based health worker	$16	Dominant	$3090	Dominant
	Effectiveness of injectable contraceptives	Dominant	Dominant	Dominated	$1339
12-month continuation rate with DMPA-IM	Direct medical costs for first DMPA-IM injection by a facility-based health worker	$74	Dominant	$2773	Dominant
	ACM effectiveness among women who discontinued self-injection of DMPA-SC	$66	Dominant	Dominated	Dominant
	Direct medical costs for each subsequent DMPA-SC self-injection away from the facility	Dominant	$317	$1224	$5307
	Direct medical costs for each subsequent DMPA-IM injection by a facility-based health worker	$69	Dominant	$3390	Dominant
	Effectiveness of injectable contraceptives	$328	Dominant	$743	$1443
Direct medical costs for each subsequent DMPA-IM injection by a facility-based health worker	ACM effectiveness among women who discontinued self-injection of DMPA-SC	$1497	$72	Dominant	Dominant
	Direct medical costs for each subsequent DMPA-SC self-injection away from the facility	$390	$1173	Dominant	$189
	Effectiveness of injectable contraceptives	Dominated	$719	$1275	Dominant
ACM effectiveness among women who discontinued self-injection of DMPA-SC	Direct medical costs for each subsequent DMPA-SC self-injection away from the facility	$120	$1006	Dominant	$439
	Direct medical costs for each subsequent DMPA-IM injection by a facility-based health worker	$1497	Dominant	$72	Dominant
	Effectiveness of injectable contraceptives	Dominated	$543	Dominant	Dominant

Definitions: The term “dominant” means that self-injection averts more DALYs and costs less than provider-administered DMPA-IM. The term “dominated” describes the opposite situation: self-injection averts less DALYs and costs more than provider-administered DMPA-IM.

**Fig. 3:**
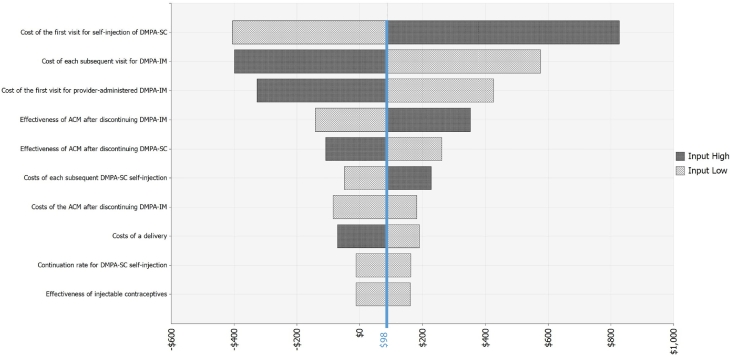
Probabilistic sensitivity analysis for the health system perspective (under program design).

We show the probabilistic sensitivity analysis results under a health system perspective in Fig. 3. In the probabilistic sensitivity analyses, the cost inputs are the top three most influential model inputs. These inputs are the costs for the first visit for self-injection of DMPA-SC and the costs for the first and subsequent visits for health-worker-administered DMPA-IM. Using the conservative threshold of $293 per DALY averted to determine whether self-injection of DMPA-SC is cost-effective compared to health-worker-administered DMPA-IM, we found that in 73% of the iterations when the one-page instruction sheet is used as the training aid, self-injected DMPA-SC is likely to be cost-effective compared to health-worker-administered DMPA-IM, while it is likely to be cost-effective in 51% of the cases if the booklet is used as the training aid. If the higher WHO cost-effectiveness thresholds are used (where an intervention is considered highly cost-effective for Uganda if the incremental cost per DALY averted is below $615 and cost-effective if between $615 and $1845), self-injection is highly cost-effective in 84% of the cases and cost-effective in 95% of the cases when the instruction sheet is used as the training aid. If the booklet is used, then self-injection is highly cost-effective in 66% of the cases and cost-effective in 90% of the cases.

## 4 Discussion

To the best of our knowledge, this is the first cost-effectiveness study of DMPA-SC self-injection relative to the more commonly available DMPA-IM administered by facility-based health workers. Our results showed that, compared to provider-administered DMPA-IM, self-injection of DMPA-SC (1) averted more pregnancies and DALYs and costed less, from a societal perspective, and (2) may be cost-effective depending on the program design, from a health system perspective. These findings were robust to variations in key model inputs. The two main drivers of the cost-effectiveness results were the costs of the first visit for women self-injecting with DMPA-SC and the effectiveness of the ACM to which women switched after discontinuing DMPA. Using the instruction sheet as the training aid makes self-injection of DMPA-SC cost-effective compared to health-worker-administered DMPA-IM in the majority of the probabilistic sensitivity analysis iterations.

Nearly 20 years ago, the United States Public Health Service's Panel on Cost-Effectiveness in Health and Medicine argued the importance of including the societal perspective in cost-effectiveness analysis, that “all parties be aware of and consider the interests of others” [32]. Put simply, analysts should consider any client preferences for more expensive interventions within the context of the benefits of these interventions for clients, in addition to the costs to the health system. This analysis attempted to account for multiple perspectives. The benefits of self-injection for family planning clients are clear. Moreover, from a health system perspective, the benefits of self-injection relative to DMPA-IM justify the incremental costs of this new delivery strategy under low-cost training programs.

The scenarios explored in this analysis reflect the fact that self-injection program design is rapidly evolving. During the research study, staff did not provide disposal containers to participants in the research study, but under program scale up, women receive disposal containers to facilitate safe medical waste disposal as part of self-injection service delivery in Uganda. Also, when we first designed this study, stakeholders in Uganda were relatively cautious about potential rollout of self-injection [33]. We carefully vetted the research intervention designed for the continuation study on self-injection in Uganda reflected in this analysis with stakeholders and family planning clients. Both groups, especially clients, preferred the self-injection instruction booklet over the shorter one-page format now being used in service delivery in Uganda. However, as various groups have become more familiar and comfortable with the concept of self-injection and more women have become comfortable as self-injectors, it has been possible to revise the program to make scale-up more affordable over the long term. In addition to replacing the booklet with the one-page instruction guide, programs in Uganda are exploring variations such as eliminating practice injections — instead having clients learn by watching health worker demonstrations — and offering self-injection training from community health workers rather than higher-level facility-based health workers. Recent results from Malawi on the provision of self-injection through community health workers are promising [20]. These latter factors may alter the cost-effectiveness of self-injection relative to DMPA-IM, but we did not explore them in this study.

The study had several limitations. First, the analysis took a 1-year time horizon, and hence, we allocated upfront costs (e.g., to train women to self-inject) in the first year. While a longer time horizon would spread the costs of self-injection training over several years, continuation rates for injectables would decrease (data not available). Second, the analysis only estimated the maternal DALYs averted and did not include neonatal DALYs; thus, we underestimated the benefits of self-injection and some health system savings. Lastly, we conducted this study under a research setting and did not account for other important programmatic costs, such as those for introduction and supply chain.
